# GSK3B inhibition reduced cervical cancer cell proliferation and migration by modulating the PI3K/Akt signaling pathway and epithelial-to-mesenchymal transition

**DOI:** 10.1590/1414-431X2024e13796

**Published:** 2024-08-19

**Authors:** Yanhong Zheng, Yang Yang, Weiyan Zhu, Ruhao Liu, Aodong Liu, Runfeng Zhang, Weixing Lei, Shifeng Huang, Yongzhu Liu, Qinglan Hu

**Affiliations:** 1 The Affiliated Qingyuan Hospital (Qingyuan People's Hospital), Guangzhou Medical University, Qingyuan, Guangdong, China; 2 Luoyuan Center for Disease Control and Prevention, Fuzhou, China

**Keywords:** Cell proliferation and migration, Cervical cancer, Epithelial-to-mesenchymal transition, Glycogen synthase kinase 3β, Phosphatidylinositol 3-carboxykinase/protein kinase B signaling pathway

## Abstract

Previous studies show that glycogen synthase kinase 3β (GSK3B) plays an important role in tumorigenesis. However, its role in cervical cancer is unclear. The present study silenced GSK3B with siRNAs and/or chemical inhibitors to determine its role in HeLa cervical cancer cell proliferation and migration as well as in xenograft tumor growth. Cell Counting Kit (CCK)-8 and 5-ethynyl-2'-deoxyuridine (EdU) assays were used to determine cell survival and proliferation. Scratch and Transwell^®^ assays were used to evaluate cell migration. Xenograft tumors were used to evaluate the effect of GSK3B on tumor growth. Transcriptomic sequencing was used to clarify the mechanisms underlying the foregoing processes. Public databases and clinical specimens showed that GSK3B was upregulated in cervical cancer tissues and correlated with poor prognosis. *In vitro* experiments indicated that GSK3B inhibition reduced cell viability, proliferation, and migration. *In vivo* experiments demonstrated that GSK3B inhibition slowed xenograft tumor growth. Transcriptomic sequencing revealed that GSK3B inhibition modulated the phosphatidylinositol 3-carboxykinase (PI3K)/protein kinase B (Akt) and extracellular matrix (ECM)-receptor interaction signaling pathways. GSK3B inhibition decreased the protein levels of phosphorylated PI3K and Akt and the levels of mesenchymal markers but increased those of epithelial markers. An activator of the PI3K/Akt signaling pathway counteracted the suppressive effects of GSK3B inhibition on HeLa cell viability and proliferation and on PI3K/Akt signaling. Our data suggested that GSK3B regulated cervical cancer cell proliferation and migration by modulating the PI3K/Akt signaling pathway and epithelial-to-mesenchymal transition (EMT).

## Introduction

Cervical cancer is a common gynecological malignancy. Advanced cases are difficult to treat and are characterized by high recurrence and mortality rates (1-3). The global incidence of cervical cancer has increased and mean patient age has decreased in recent years (4). Therefore, it is necessary to elucidate the mechanisms of cervical cancer onset and progression and explore novel therapeutic targets and strategies.

Glycogen synthase kinase 3β (GSK3B) is a conserved serine/threonine (Ser/Thr) kinase that inhibits glycogen synthase in resting cells (5). GSK3B plays key roles in cell proliferation, DNA repair, cell signal transduction, and metabolic pathways (6). GSK3B dysregulation is linked to certain cancers (7), lipid metabolic disorders (8), and Alzheimer's disease (9).

GSK3B inhibits certain tumors by modulating the Wnt/β-catenin signaling pathways. However, it may also function as a tumor promoter (10). GSK3B overexpression drives proliferation, suppresses apoptosis, and promotes invasion of pancreatic ductal adenocarcinoma (PDAC) cells (11). Wu et al. (12) reported that TNF receptor-associated factor 6 (TRAF6) could lead to autophagic degradation of oncogenic β-catenin (CTNNB1) and overexpression of GSK3B in colorectal cancer (CRC) causing degradation of TRAF6, finally increasing the β-catenin level and promoting CRC metastasis. GSK3B inhibitors suppressed xenograft tumor growth in several mouse models. A dual inhibitor targeting both GSK3B and histone deacetylases (HDACs) suppressed pancreatic tumor growth and metastasis in mice (13). GSK3B inhibitors also worked synergistically with paclitaxel to suppress non-small cell lung cancer (NSCLC) cells (14). Nevertheless, the role of GSK3B in cervical cancer is poorly understood, and it is unknown whether GSK3B inhibitors are efficacious in cervical cancer treatment.

In the present work, we knocked down GSK3B and/or chemically inhibited it in HeLa cells and in xenograft tumors to clarify its functions in cervical cancer. We also investigated the mechanisms by which it modulates cell survival, proliferation, and migration.

## Material and Methods

### Ethical statement

Examination of GSK3B expression in clinical cervical cancer samples was approved by Qingyuan People's Hospital Medical Ethics Committee (IRB-2024-063; https://www.medicalresearch.org.cn/login).

### Cell culture

HeLa cells were cultured in Dulbecco's modified Eagle's medium (DMEM; Corning Life Sciences, USA) supplemented with 10% fetal bovine serum (FBS; Gibco, USA) and 1% (w/v) antibiotic mixture (Beyotime Biotechnology, China) in a 5% CO_2_ incubator at 37°C. When the cells reached 100% confluence, they were digested with 0.25% (w/v) trypsin (Gibco) to generate a single-cell suspension. An appropriate number of cells was then transferred to a fresh complete medium and maintained in a 5% CO_2_ incubator at 37°C.

### Cell treatment

A Cell Counting Kit (CCK)-8 or 5-ethynyl-2'-deoxyuridine (EdU) assay was performed after an RNA interference assay for 48 h to determine the effect of GSK3B knockdown on HeLa cell viability and proliferation. HeLa cells subjected to a 24-h RNA interference assay were seeded onto new plates, and a scratch or Transwell^®^ cell migration assay was performed at various time points to determine the effect of GSK3B knockdown on HeLa cell migration. HeLa cells were subjected to a 48-h RNA interference assay and a western blot (WB) assay was performed to determine the effect of GSK3B knockdown on signaling pathways.

HeLa cells subjected to a 48-h RNA interference assay were exposed to 10 μM of the PI3K activator 740 Y-P (MedChemExpress, USA) for 24 h, and a CCK-8, EdU, or WB assay was performed to determine the effects of GSK3B knockdown and 740 Y-P treatment on HeLa cell viability, proliferation, and signaling pathways.

A CCK-8 or EdU assay was performed on HeLa cells treated with 0.5 μM of the GSK3B inhibitor CP21R7 (MedChemExpress) for 48 h to determine the effect of GSK3B inhibition on HeLa cell viability and proliferation. A scratch or Transwell^®^ cell migration assay was then performed on the HeLa cells exposed to 0.5 μM CP21R7 to determine the effect of GSK3B inhibition on cell migration at various time points.

HeLa cells were subjected to 0.5 μM CP21R7 plus 10 μM 740 Y-P, and CCK-8, EdU, and WB assays were used to determine the effect of GSK3B inhibition plus 740 Y-P treatment on HeLa cell viability, proliferation, and signaling pathways.

### RNA interference assay

HeLa cells in the growth phase were seeded onto six-, 24-, and 96-well plates at densities of 5×10^5^, 0.8×10^5^, and 1.5×10^4^/well, respectively, and cultured until they reached 60-80% confluence. For transfection, 200, 100, and 50 μL serum-free culture medium and 100, 25, and 12.5 pmol siRNA (GenePharma, China), respectively, were mixed in a 1.5-mL centrifuge tube and 10, 2.5, and 1.25 μL transfection reagent (GenePharma), respectively, were added to the mixtures. The latter were blended thoroughly, left to stand for 10 min, and added to the six-, 24-, and 96-well plates. The sequences of the siRNAs targeted GSK3B are listed in Table 1.

**Table 1:** Sequences of the siRNA-1 and siRNA-2 used in the present study to target GSK3B.

siRNA-1(#1)-sense	GCU AGA UCA CUG UAA CAU ATT
siRNA-1(#1)-antisense	UAU GUU ACA GUG AUC UAG CTT
siRNA-2(#2)-sense	GGA AAC AGU AUA CAG AGU UTT
siRNA-2(#2)-antisense	AAC UCU GUA UAC UGU UUC CTT

### CCK-8 assay

HeLa cells were cultured in a 96-well plate at a density of 1.5×10^4^/well for RNA interference and at a density of 5×10^3^/well for treatment either with CP21R7 alone or with CP21R7 plus 740 Y-P. Then, 10 μL of the CCK-8 reagent (MedChemExpress) was added to each well and the plates were incubated in the dark for 1 h. Absorbance was measured with an enzyme-linked immunosorbent assay (ELISA) reader to calculate the changes in HeLa cell viability.

### EdU assay

HeLa cells were seeded onto a 24-well plate at a density of 0.8×10^5^/well and their proliferation was detected with a commercial kit (No. C10310; Guangzhou RiboBio Co. Ltd., China) according to the manufacturer's instructions.

### Western blot (WB) assay

HeLa cells were seeded onto a six-well plate at a density of 5×10^5^/well and cultured overnight. After related treatment, cells were lysed in radioimmunoprecipitation assay (RIPA) buffer on ice. The cell lysate was then collected and centrifuged at 10,629 *g* and 4°C for 15 min, the supernatant was collected, and its protein concentration was determined with a bicinchoninic acid (BCA) kit (Beyotime Biotechnology). Thirty micrograms of this protein solution were mixed with 5× loading buffer at a 4:1 (v/v) ratio, denatured at 100°C for 5 min, and electrophoresed on a precast gel. The proteins were then transferred to a polyvinylidene fluoride (PVDF) membrane at 400 mA for 1 h. The membrane was blocked with 5% (w/v) skim milk at room temperature (RT) for 1 h and incubated with the primary antibodies anti-GSK3B (No. 3D10; Cell Signaling Technology (CST), USA), anti-p-PI3K (No. E3U1H; CST), anti-p-AKT (No. D9E; CST), anti-fibronectiin (No. ab268020; Abcam, UK), anti-N-cadherin (No. A19083; ABclonal, USA), anti-E-cadherin (No. A22850; ABclonal), and anti-GAPDH (No. MC4; Beijing Ray Antibody Biotech, China) at 4°C overnight. The membrane was then incubated with a goat anti-mouse IgG(H+L)-HRP (RM3001L, Beijing Ray Antibody Biotech, China) or goat anti-rabbit IgG(H+L)-HRP secondary antibody (RM3002L, Beijing Ray Antibody Biotech, China) at RT for 1 h and subjected to enhanced chemiluminescence (ECL) detection in a gel imaging system.

### Scratch assay

HeLa cells were seeded onto a six-well plate at a density of 1.5×10^6^/well. At 100% confluence, the cell layer was scratched with a sterile pipette tip, and detached cells were rinsed off with phosphate-buffered saline (PBS). The plate was then photographed under a microscope at 0, 24, 48, and 72 h and the cell migration rates were calculated.

### Transwell^®^ cell migration assay

A Transwell^®^ cell migration chamber (Corning Life Sciences) was placed in a 24-well plate. HeLa cells were digested into a single-cell suspension and added to a serum-free medium at a density of 2.5×10^4^/mL. Then, 300 μL of the HeLa cell suspension and 750 μL of complete culture medium were added to the upper and lower Transwell^®^ cell migration chambers, respectively. The 24-well plate was incubated for 12 h and the cells were fixed with 4% (v/v) paraformaldehyde (PFA) at RT for 15 min, stained with 0.1% (w/v) crystal violet at RT for 30 min, and washed thrice with distilled water. Unmigrated cells were removed with cotton swabs and the migrated cells were photographed and enumerated under a light microscope (Sunny Optical Technology, China).

### Transcriptomic sequencing

HeLa cells were subjected to RNA interference for 48 h, lysed in RNA extraction reagent (No. AG21101; Accurate Biotechnology Co. Ltd.), and subjected to transcriptomic sequencing by Tsingke Biotech, China.

### Immunofluorescence assay (IF)

Briefly, slides were deparaffinized and rehydrated, followed by antigen retrieval with sodium citrate buffer. Then, slides were blocked with 10% goat serum for one hour at room temperature, followed by incubation with the primary antibodies at 4°C overnight. Subsequently, slides were incubated with fluorescein-conjugated secondary antibody for one hour at RT. Lastly, photographs were taken with a laser scanning confocal microscope (Carl Zeiss, Germany). The fluorescence intensity was analyzed by ImageJ software (NIH, USA).

### *In vivo* tumorigenesis

The Institutional Animal Care and Use Committee (Qingyuan People Hospital Medical Ethics Committee, China) approved the animal experiment. Specifically, 4-week-old female BALB/c nude mice (n=5) (Guangdong Medical Laboratory Animal Center, China) were subcutaneously implanted with HeLa cells (1×10^6^ cells/mouse). Tail vein injection of CP21R7 or equivalent DMSO (5 mg/kg) was performed at day 7 after implantation with HeLa cells. The tumor's width, length, and height were measured by a vernier caliper, and tumor volume was assessed every 3 days. Tumor volume was calculated using the formula π/6 × width × length × height (mm^3^). After a 25-day interval, the mice were euthanized and xenograft tumors were collected.

### Statistical analysis

Data were processed in GraphPad Prism v. 8.4.2 (GraphPad Software, USA). Unpaired *t*-tests were used for pairwise group comparisons. Data are reported as means±SE.

## Results

### GSK3B upregulation was correlated with poor cervical cancer prognosis

**Figure 1:**
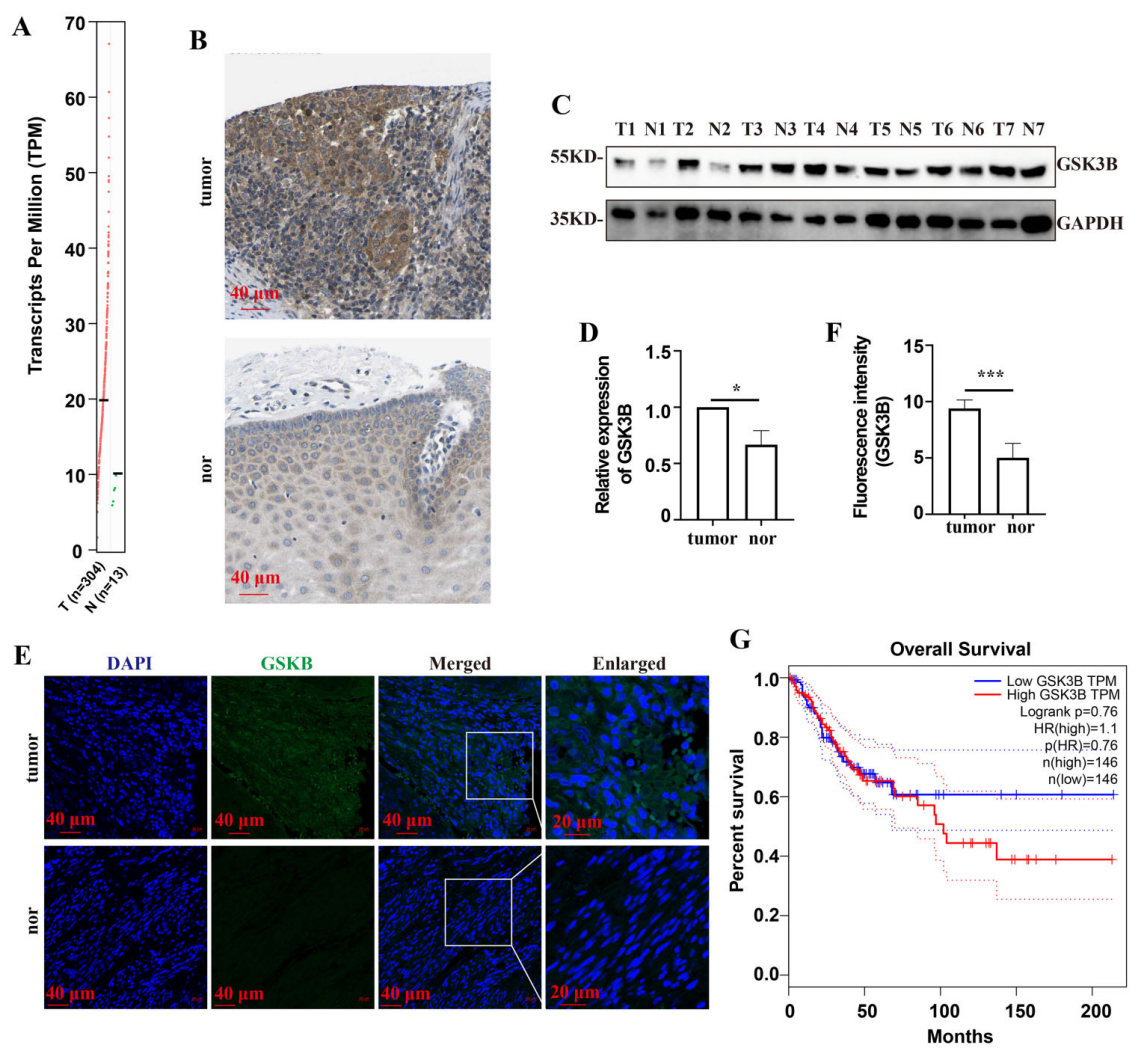
Relationship between GSK3B expression and cervical cancer prognosis. **A**, GEPIA data analysis showing GSK3B expression in cervical cancer (T) and normal (N) paratumor tissues. **B**, Representative immunohistochemical results of GSK3B expression in cervical cancer (tumor) and normal (nor) paratumor tissues (scale bar 40 μm) from the HPA database. **C**, Representative western blot results of GSK3B expression in cervical cancer (T) and normal (N) paratumor tissues from clinical specimens and (**D**) the corresponding statistical analysis. **E**, Representative immunofluorescence assay results of GSK3B expression in cervical cancer (tumor) and normal (nor) paratumor tissues from clinical specimens (scale bar 40 and 20 μm) and (**F**) the corresponding statistical analysis. **G**, Survival analysis based on the GEPIA database showing that patients with cervical cancer and high GSK3B expression have poorer prognosis than those with low GSK3B expression. Data are reported as means±SE. *P<0.05, ***P<0.001 (*t*-test).

The Gene Expression Profiling Analysis (GEPIA) database (http://gepia.cancer-pku.cn/) revealed that the GSK3B expression levels were higher in cervical cancer tissues than in normal paratumor tissues (Figure 1A). The Human Protein Atlas database (https://www.proteinatlas.org/) showed that the GSK3B protein levels were higher in cervical cancer tissues than in normal paratumor tissues (Figure 1B). We also examined the expression of GSK3B in cervical cancer specimens. In line with the results from databases, both the WB analysis (Figure 1C and D) and IF analysis (Figure 1E and F) showed that GSK3B expression was higher in cervical cancer tissues than in normal paratumor tissues. The GEPIA database also disclosed that patients with high GSK3B expression levels had shorter survival times than those with low GSK3B expression levels (Figure 1G). The preceding findings suggested that GSK3B was implicated in cervical cancer tumorigenesis and that the prognosis was poorer for patients with high GSK3B levels.

### GSK3B inhibition reduced HeLa cell survival and proliferation

**Figure 2:**
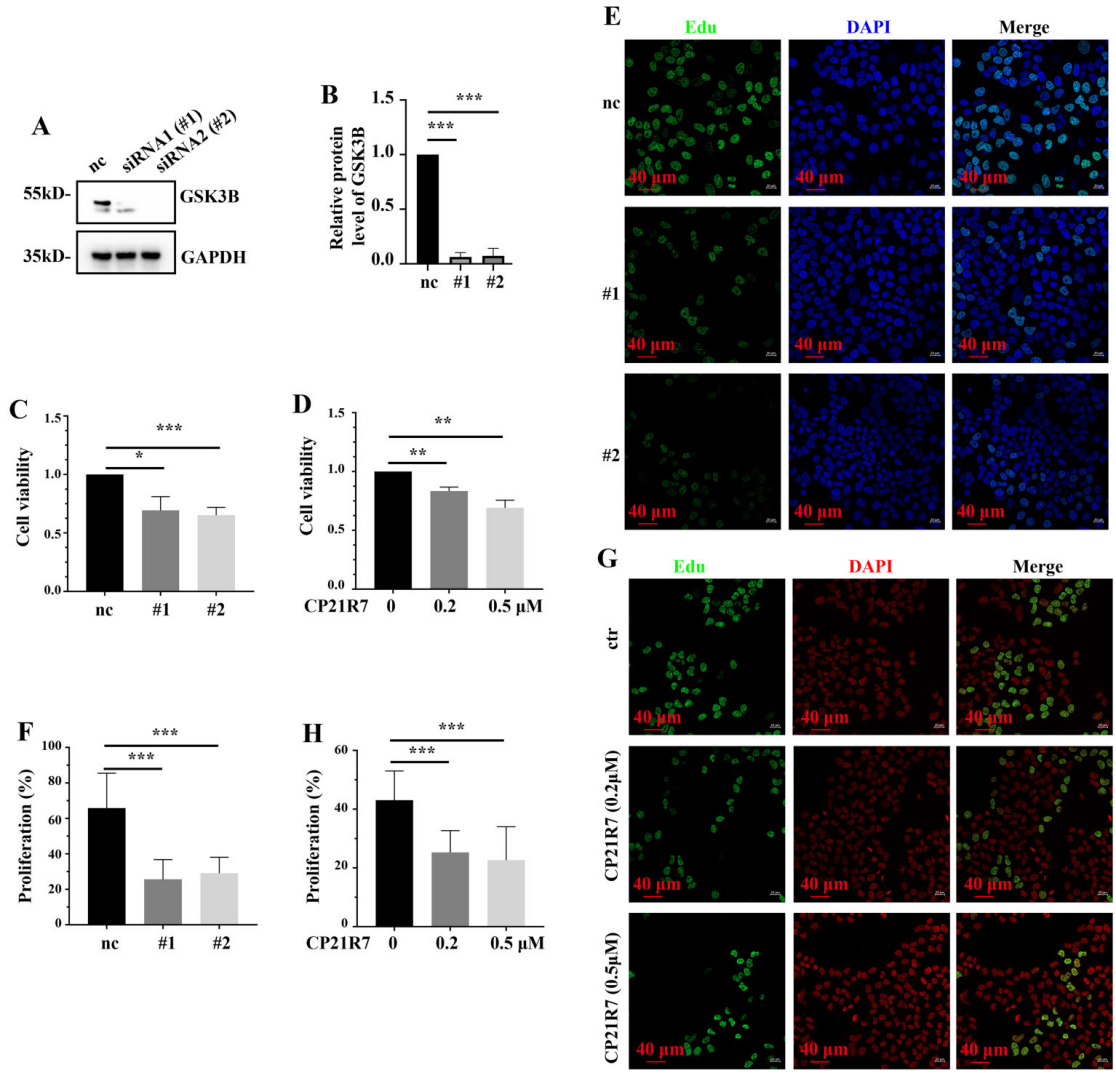
GSK3B suppression reduced HeLa cell viability and proliferation. Western blot analysis of GSK3B siRNA efficacy (**A**) and the corresponding statistical analysis (**B**). Statistical analysis of CCK-8 assay of the viability of HeLa cells with GSK3B knockdown (**C**) or CP21R7 treatment (**D**). EdU assay showing the proliferation of HeLa cells with GSK3B knockdown (**E**) or CP21R7 treatment (**G**) (scale bar 40 μm) and the corresponding statistical analyses (**F** and **H**). Data are reported as means±SE. *P<0.05, **P<0.01, ***P<0.001 (ANOVA). nc: negative control. #1 and #2: See Table 1 for sequences of the siRNA-1 and siRNA-2 used to target GSK3B.

We knocked down GSK3B (Figure 2A and B) or inhibited its activity with CP21R7 in HeLa cells to verify its oncogenic role in cervical cancer. Both GSK3B knockdown and the CP21R7 treatment significantly lowered HeLa cell viability (Figure 2C and D). An EdU assay revealed that siRNAs and CP21R7 reduced HeLa cell proliferation as well (Figure 2E-H). Hence, GSK3B inhibition may lower the viability of HeLa cells by reducing their proliferation.

### GSK3B inhibition suppressed HeLa cell migration

**Figure 3:**
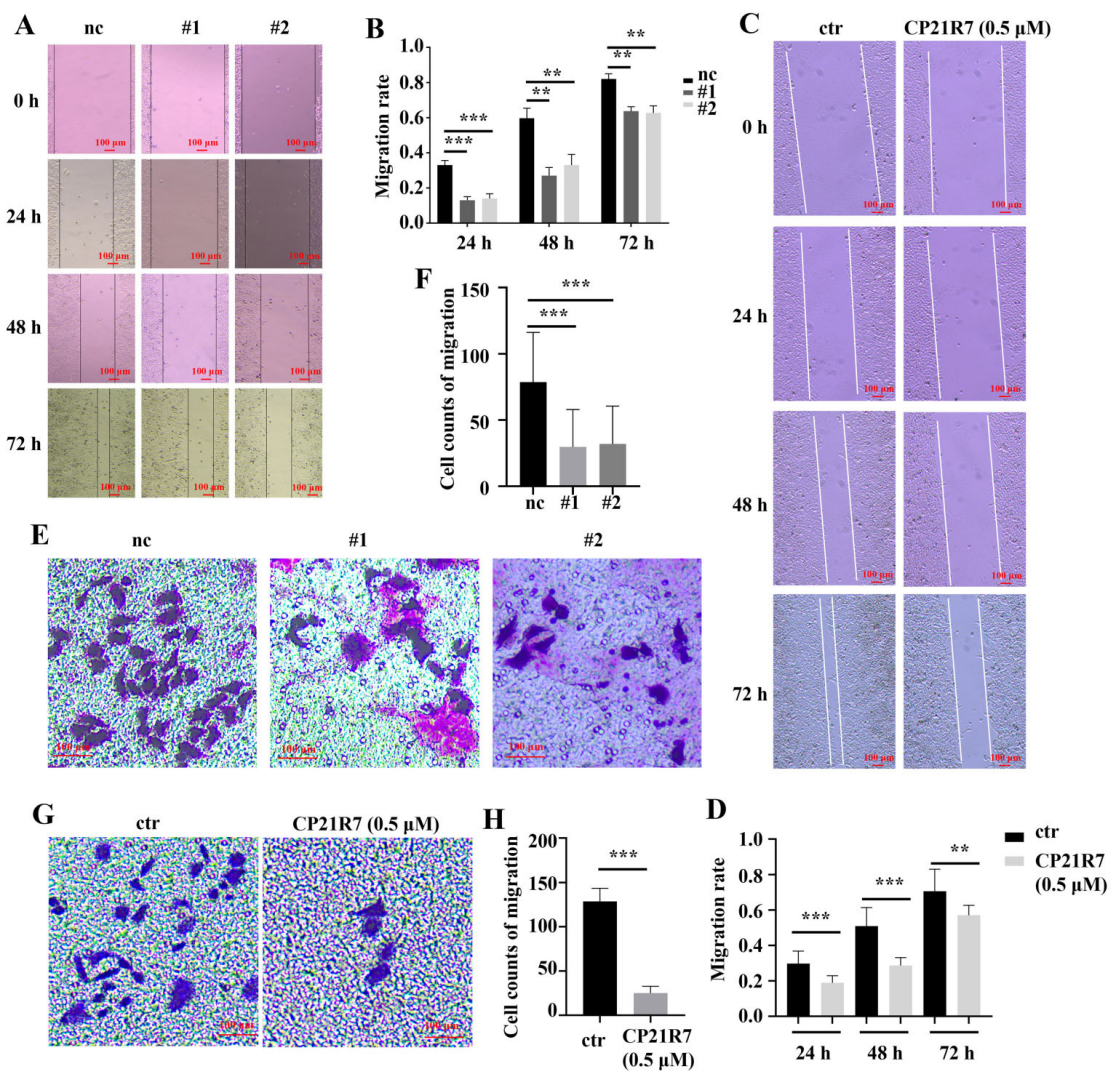
GSK3B suppression reduced HeLa cell migration. Scratch assay showing migration in HeLa cells with GSK3B knockdown (**A**) or CP21R7 treatment (**C**) at various time points and the corresponding statistical analyses (**B** and **D**). The Transwell^®^ cell migration assay detecting migration in HeLa cells with GSK3B knockdown (**E**) or CP21R7 treatment (**G**) and the corresponding statistical analyses (**F** and **H**). Data are reported as means±SE. **P<0.01, ***P<0.001 (ANOVA). **A**, **C**, **E**, and **G** scale bar 100 μm. ctr: control. #1 and #2: See Table 1 for sequences of the siRNA-1 and siRNA-2 used to target GSK3B.

The scratch assay revealed that both siRNAs and CP21R7 significantly reduced HeLa cell migration at 24, 48, and 72 h (Figure 3A-D). The Transwell^®^ cell migration assay disclosed that both siRNAs and CP21R7 significantly reduced the number of migrated HeLa cells (Figure 3E-H). Taken together, the results demonstrated that GSK3B inhibition suppressed HeLa cell migration.

### GSK3B inhibition suppressed tumor growth in xenograft models

**Figure 4:**
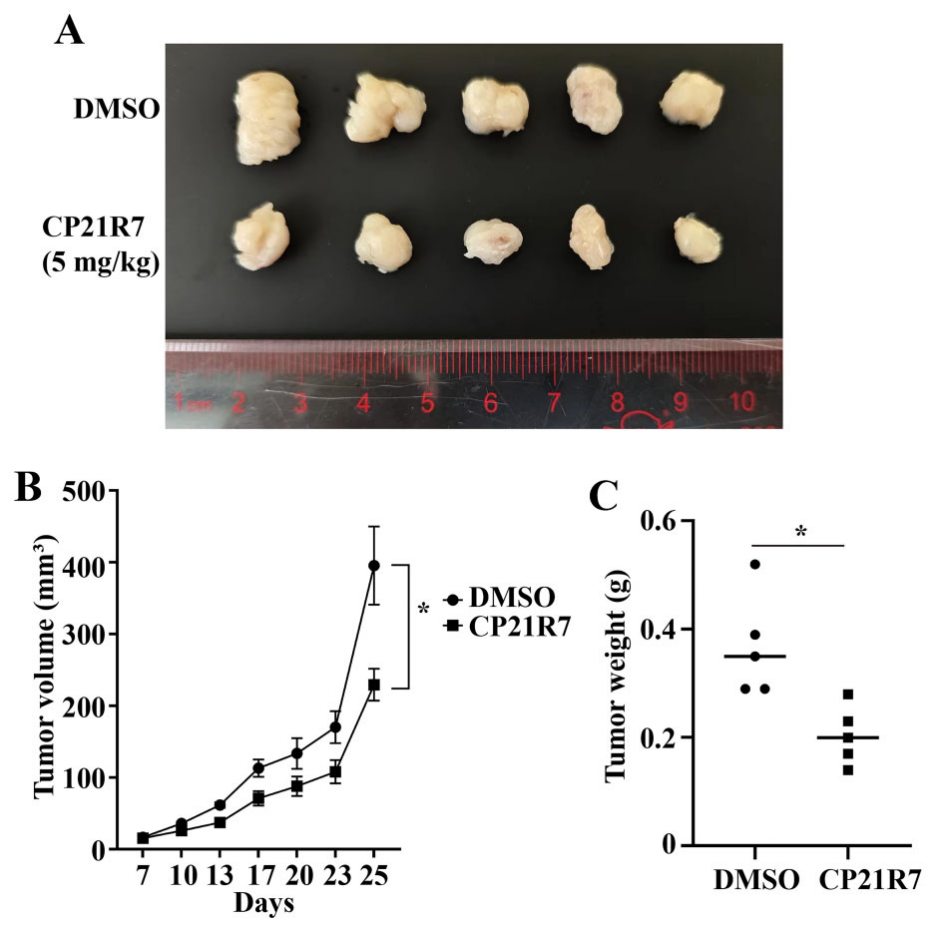
Inhibition of GSK3B slowed the tumor growth in mouse models. **A**, Photograph of xenograft tumors at study endpoint. **B**, Tumor growth curve. **C**, Tumor weight analysis. Data are reported as means±SE. *P<0.05 (*t*-test).

To further investigate the oncogenic role of GSK3B in cervical cancer, we performed an *in vivo* experiment in nude mice. As shown in the results, mice injected with CP21R7 showed slower xenograft tumor growth (Figure 4). Therefore, these data demonstrated the critical role of GSK3B in controlling the tumorigenesis of cervical cancer.

### Transcriptomic sequencing identified potential gene targets in the GSK3B-mediated regulation of HeLa cell proliferation and migration

**Figure 5:**
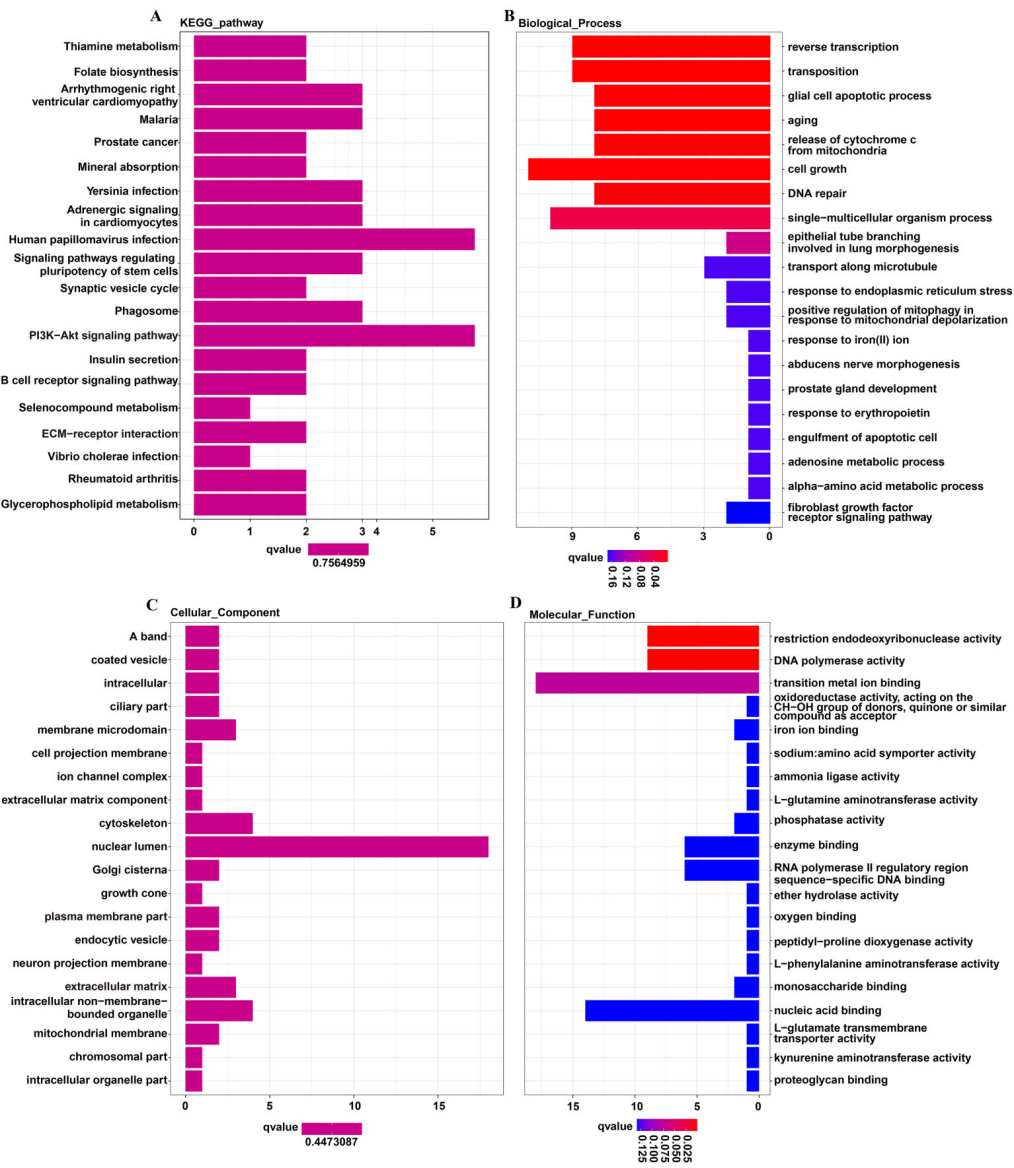
Transcriptomic sequencing showing the target genes and signaling pathways regulated by GSK3B. **A**, KEGG analysis showing the top 20 pathways enriched in differentially expressed genes (DEGs). **B**, GO analysis showing the top 20 enriched biological process terms. **C**, GO analysis of cellular components enriched in DEGs. **D**, GO analysis of molecular functions enriched in DEGs.

We knocked down GSK3B in HeLa cells and performed transcriptomic sequencing on them to elucidate the mechanisms by which GSK3B drives cervical cancer progression. As shown in the results, the PI3K/Akt and ECM-receptor interaction signaling pathways were highly enriched (Figure 5A). A gene ontology (GO) analysis showed that proliferative genes regulating GSK3B in cellular processes were highly enriched biological processes (Figure 5B). The cellular component distributions (Figure 5C) and the molecular functions (Figure 5D) of these targets are illustrated by a bar chart.

### GSK3B inhibition inactivated PI3K/Akt signaling

**Figure 6:**
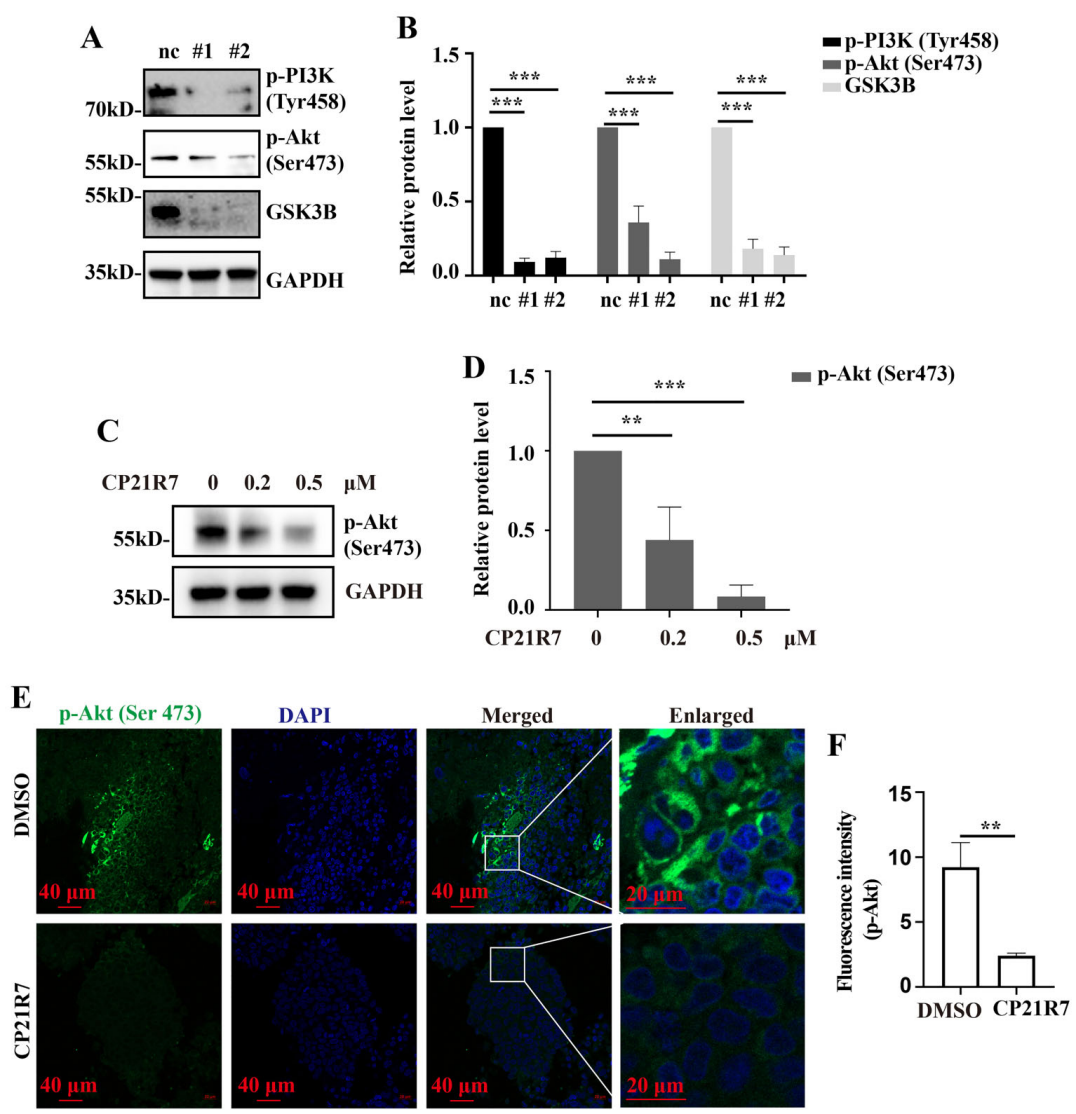
Inhibition of GSK3B inactivates PI3K/Akt signaling. Western blot (WB) analysis detecting the p-PI3K and p-Akt protein levels in HeLa cells with GSK3B knockdown (**A**). WB analysis detecting the p-Akt protein levels in HeLa cells treated with CP21R7 (**C**) and the corresponding statistical analyses (**B** and **D**). **E**, Immunofluorescence assay analysis detecting the p-Akt protein levels in xenograft tumors (scale bar 40 or 20 μm) and the corresponding statistical analyses (**F**). Data are reported as means±SE. **P<0.01, ***P<0.001 (ANOVA). nc: negative control. #1 and #2: See Table 1 for sequences of the siRNA-1 and siRNA-2 used to target GSK3B.

The PI3K/Akt signaling pathway regulates cell proliferation, survival, metabolism, and angiogenesis (15) and its overactivation is usually associated with tumor development ([Bibr B16] [Bibr B17] 1618). Several PI3K/Akt inhibitors are administered as cancer treatments (19,20). Transcriptomic sequencing demonstrated that PI3K/Akt signaling was highly enriched in the differentially expressed genes (DEGs) between GSK3B-knockdown and normal HeLa cells (Figure 5A). We then used WB analysis to verify the impact of GSK3B knockdown on PI3K/Akt signaling and found that it significantly decreased the phosphorylation of PI3K (p-PI3K) and Akt (p-AKT) in HeLa cells (Figure 6A and B). The CP21R7 treatment also reduced p-AKT (Figure 6C and D). In addition, CP21R7 treatment decreased the p-AKT level in the *in vivo* xenograft tumor (Figure 6E and F). Thus, GSK3B might regulate cervical cancer cell proliferation and survival by modulating the PI3K/Akt signaling pathway.

### GSK3B inhibition reversed the EMT phenotype

**Figure 7:**
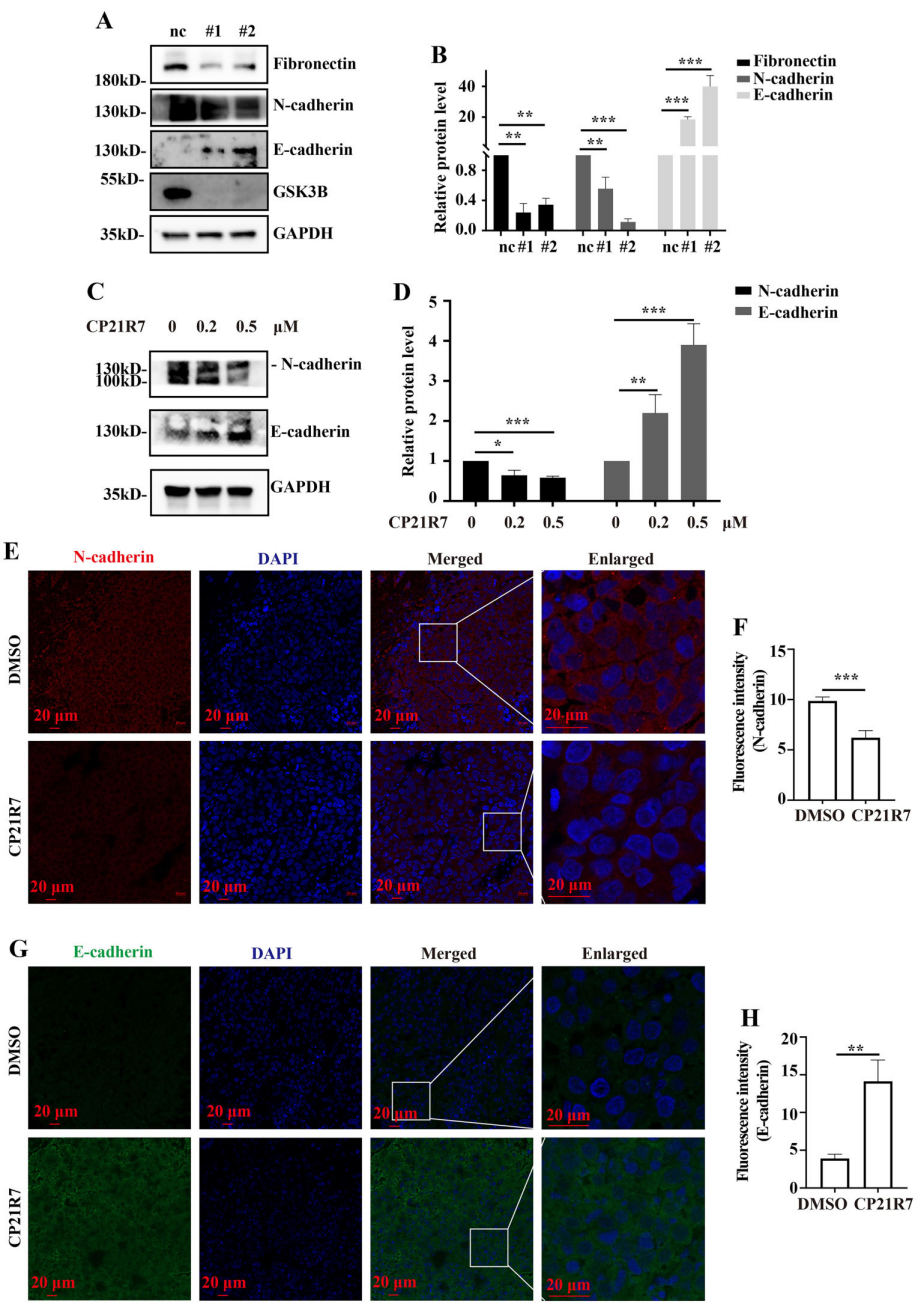
GSK3B inhibition suppressed epithelial-to-mesenchymal transition (EMT). Western blot analysis of fibronectin, N-cadherin, and E-cadherin in HeLa cells with GSK3B knockdown (**A**) and of N-cadherin and E-cadherin protein levels in HeLa cells treated with CP21R7 (**C**) with the corresponding statistical analyses (**B** and **D**). Fluorescence intensity analysis of N-cadherin protein levels (**E**) and E-cadherin protein levels (**G**) in xenograft tumors (scale bar 200 μm) with the corresponding statistical analyses (**F** and **H**). Data are reported as means±SE. *P<0.05, **P<0.01, ***P<0.001 (ANOVA). nc: negative control. #1 and #2: See Table 1 for sequences of the siRNA-1 and siRNA-2 used to target GSK3B.

EMT is the weakening of adhesion between epithelial cells and the basement membrane and the subsequent transformation of epithelial cells into mesenchymal cells. The latter have greater motility and migration capacity than the former (21,22). EMT activation is closely associated with tumor metastasis (23). Here, transcriptomic sequencing disclosed that the ECM-receptor interaction signaling pathway was enriched in the DEGs between GSK3B-knockdown and normal HeLa cells. WB analysis showed that GSK3B knockdown significantly decreased the protein levels of the mesenchymal markers fibronectin and N-cadherin and increased the protein level of the epithelial marker E-cadherin in HeLa cells (Figure 7A and B). Similarly, the CP21R7 treatment decreased N-cadherin but increased the protein levels of E-cadherin in *in vitro* experiments (Figure 7C and D) and in *in vivo* xenograft tumors (Figure 7E-H). Thus, GSK3B inhibition promoted the transition of HeLa cells from a mesenchymal-like state with strong migration ability to an epithelial-like state with weak migration ability. Hence, GSK3B may regulate HeLa cell migration by modulating the EMT.

### PI3K/Akt signaling pathway activation rescued the suppression of HeLa cell proliferation caused by GSK3B downregulation

**Figure 8:**
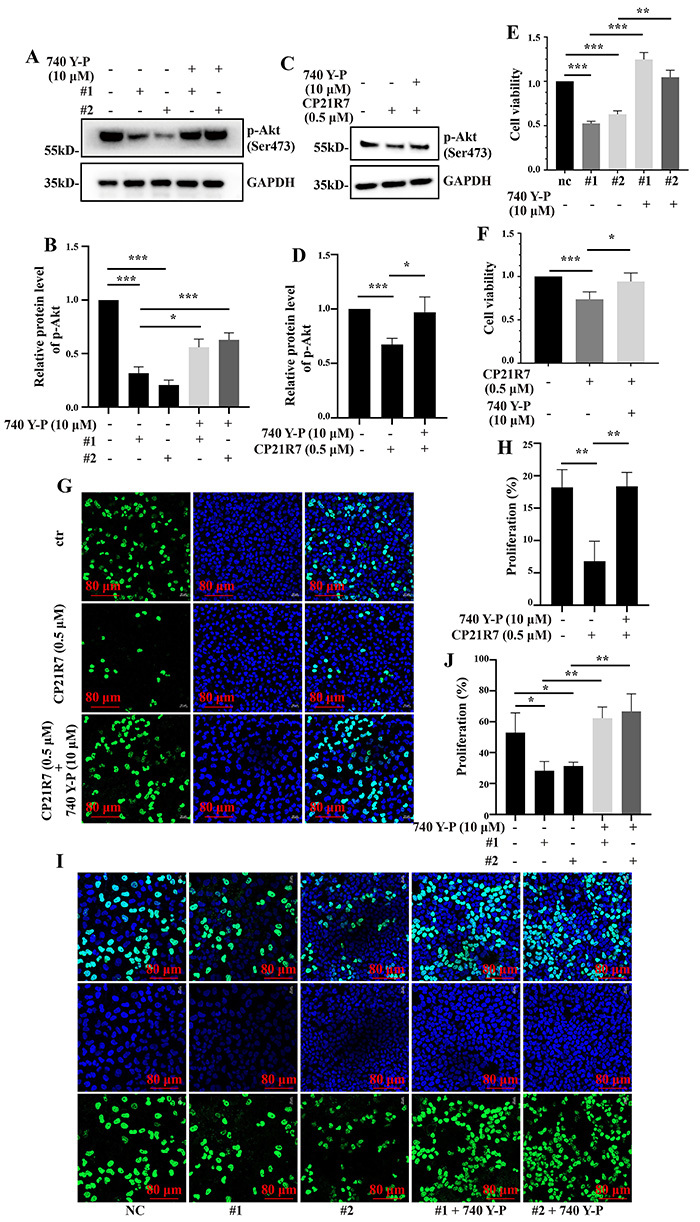
740 Y-P counteracted reductions in Akt phosphorylation and HeLa cell viability and proliferation. Western blot analysis of Akt phosphorylation in HeLa cells co-treated with GSK3B knockdown and 740 Y-P (**A**) or with CP21R7 and 740 Y-P (**C**) and the corresponding statistical analyses (**B** and **D**). Statistical analysis of a CCK-8 assay showing the viability of HeLa cells co-treated with GSK3B knockdown and 740 Y-P (**E**) or with CP21R7 and 740 Y-P (**F**). EdU assay showing the proliferation of HeLa cells co-treated with CP21R7 and 740 Y-P (**G**) or with GSK3B knockdown and 740 Y-P (**I**) (scale bar 80 μm) and the corresponding statistical analyses (**H** and **J**). Data are reported as means±SE. *P<0.05, **P<0.01, ***P<0.001 (ANOVA). nc: negative control. #1 and #2: See Table 1 for sequences of the siRNA-1 and siRNA-2 used to target GSK3B.

We applied the PI3K activator 740 Y-P to validate the role of PI3K/Akt signaling in GSK3B-mediated regulation of HeLa cell viability and proliferation. The 740 Y-P co-treatment reversed the inhibition of Akt phosphorylation in HeLa cells subjected to GSK3B knockdown or CP21R7 treatment (Figure 8A-D). It also counteracted the suppressive effect of GSK3B knockdown or inhibition on HeLa cell viability (Figure 8E and F) and proliferation (Figure 8G-J). These results indicated that GSK3B promoted HeLa cell viability and proliferation by activating PI3K/Akt signaling pathway.

## Discussion

Cervical cancer is the fourth leading cause of cancer-related deaths in women after breast, colorectal, and lung cancers (24). The incidence of cervical cancer is increasing in developing countries and the disease is affecting increasingly younger populations (4,25). Late-stage cervical cancer is difficult to treat and its recurrence and mortality rates are high. Therefore, it is necessary to clarify the mechanisms of the onset and progression of cervical cancer and identify and implement novel efficacious therapeutic targets for it.

GSK3B regulates glycogen biosynthesis. It also modulates the structural proteins and transcription factors (TFs) in the β-catenin (CTNNB1) and other signaling pathways. GSK3B affects cancer cell proliferation, differentiation, and apoptosis and participates in tumor development (26). GSK3B may either promote or inhibit tumorigenesis (26). It suppresses tumors by phosphorylating CTNNB1 and inactivating the carcinogenic Wnt/β-catenin signaling pathway (27,28). It may also be oncogenic. Grassilli et al. (29) reported that GSK3B induced resistance to the necrotizing effect of certain antitumor drugs in p53-deficient colorectal cancer (CRC) cells whereas GSK3B knockdown had the opposite effect. GSK3B overexpression prevents the degradation of oncogenic CTNNB1 and promotes metastasis in CRC (12). GSK3B inhibitors demonstrated therapeutic efficacy in various mouse xenograft tumor models. A dual inhibitor targeting GSK3B and histone deacetylases (HDACs) slowed tumor growth in mouse pancreatic and ovarian cancer models (13). GSK3B inhibitors combined with poly ADP ribose polymerase (PARP) inhibitors and paclitaxel exhibited synergistic antitumor efficacy (14,30).

However, the role of GSK3B in cervical cancer is unclear. Here, we investigated the function of GSK3B in HeLa cell tumorigenesis and *in vivo* xenograft tumor growth by gene silencing or chemical inhibition. GSK3B suppression reduced the viability, proliferation, and migration of HeLa cells. In addition, inhibition of GSK3B by CP21R7 slowed tumor growth in xenograft models. Moreover, both the database analysis and clinical specimen analysis also showed that GSK3B was upregulated in cervical cancer tissues and high GSK3B expression indicated a poor prognosis. Hence, these data confirmed an oncogenic role of GSK3B in cervical cancer.

The PI3K/Akt signaling pathway regulates cell growth, proliferation, and survival and is closely associated with the development of cancers and other diseases. PI3K/Akt inhibitors such as BKM120 are administered for the clinical treatment of certain cancers (31,32). Here, we observed that GSK3B suppression inactivated the PI3K/Akt signaling pathway by reducing the phosphorylation of both PI3K and Akt. Our transcriptomic sequencing data indicated that the DEGs targeted by GSK3B were significantly enriched in the PI3K/Akt signaling pathway and in the cell growth biological processes. The PI3K/Akt signaling pathway activator 740 Y-P counteracted the inhibitory effect of GSK3B suppression on HeLa cell viability and proliferation. Therefore, GSK3B suppression reduced cervical cancer cell viability and proliferation by inactivating the PI3K/Akt signaling pathway. As GSK3B acts downstream of the PI3K/Akt signaling pathway during cellular signal transduction, Akt can inactivate GSK3B by phosphorylating its Ser-9 residue ([Bibr B33] [Bibr B34] 3335). Therefore, a feedback loop may exist between PI3K/Akt and GSK3B, and the latter may function as a negative upstream regulator of the PI3K/Akt signaling pathway. Further research is required to elucidate the mechanisms by which GSK3B inactivates PI3K/Akt signaling.

EMT was first detected in embryogenesis and tissue self-repair (36). In EMT, the markers of epithelial cells are downregulated and the latter acquire mesenchymal characteristics such as the ability to migrate and invade (37). EMT also plays a significant role in cancer metastasis (38,39). In the present work, we observed that GSK3B knockdown and the CP21R7 treatment significantly increased the protein level of the epithelial marker E-cadherin, which is in line with a previous finding that GSK3B inhibition stabilized E-cadherin expression in breast cancer (40). Knockdown of GSK3B resulted in the downregulation of the expression of mesenchymal markers fibronectin and N-cadherin. However, CP21R7 treatment did not alter the fibronectin protein level (data not shown) but downregulated N-cadherin protein. It remains to be established whether targeting CP21R7 specificity is less effective than using anti-GSK3B siRNAs or whether it explains the differences between these treatments in terms of their impact on fibronectin expression. Furthermore, CP21R7 also decreased the N-cadherin level and increased the E-cadherin level in xenograft tumors. Therefore, combining the transcriptomic sequencing showed that the ECM-receptor interaction signaling pathway is highly enriched in the DEGs targeted by GSK3B, suggesting that GSK3B may enhance HeLa cell migration by promoting EMT.

**Figure 9:**
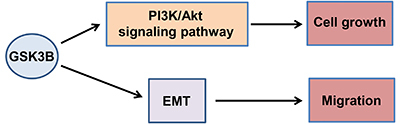
Schematic diagram of the oncogenic mechanism of GSK3B in cervical cancer. EMT: epithelial-to-mesenchymal transition.

Overall, the results of the present study suggested that GSK3B could promote cell proliferation by activating the PI3K/Akt signaling pathway and promote cell migration by inducing EMT, indicating an oncogenic role of GSK3B in cervical cancer. Thus, GSK3B inhibitors may be novel therapeutic modalities against cervical cancer (Figure 9).
